# Supplementary material for: The optimal spatially-dependent control measures to effectively and economically eliminate emerging infectious diseases
Source: PLoS Comput Biol. 2024 Oct 7;20(10):e1012498. doi: 10.1371/journal.pcbi.1012498 (PMC11486435; doi:10.1371/journal.pcbi.1012498)
Supplement: S1 Fig — Blue dots are observations and blue lines are model fitting values. Blue shaded areas are credible intervals. (PDF) [file pcbi.1012498.s001.pdf]

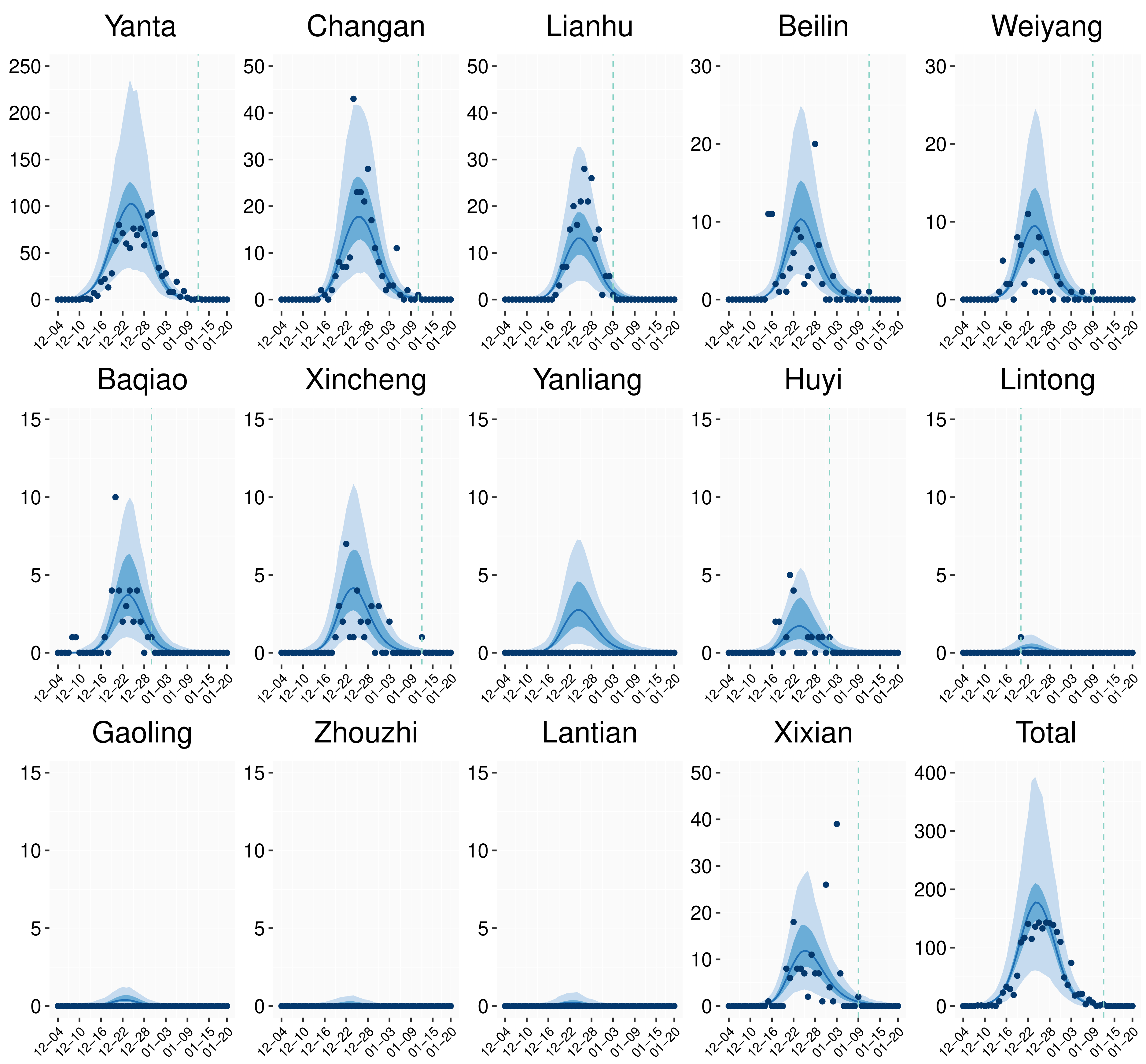

Yanta
Changan
Lianhu
Beilin
Weiyang
Baqiao
Xincheng
Yanliang
Huyi
Lintong
Gaoling
Zhouzhi
Lantian
Xixian
Total
12–04
12–10
12–16
12–22
12–28
01–03
01–09
01–15
01–20
