# Supplementary material for: The optimal spatially-dependent control measures to effectively and economically eliminate emerging infectious diseases
Source: PLoS Comput Biol. 2024 Oct 7;20(10):e1012498. doi: 10.1371/journal.pcbi.1012498 (PMC11486435; doi:10.1371/journal.pcbi.1012498)
Supplement: S2 Fig — The number of infected individuals Ii(t) under the optimal control scheme shown in Fig 3 bottom panel. Black and green vertical dotted lines indicates the time when Ii(t) and the number of new confirmed cases reaches zero. A region without a green line indicates that the number of new confirmed cases in that region is always zero. (PDF) [file pcbi.1012498.s002.pdf]

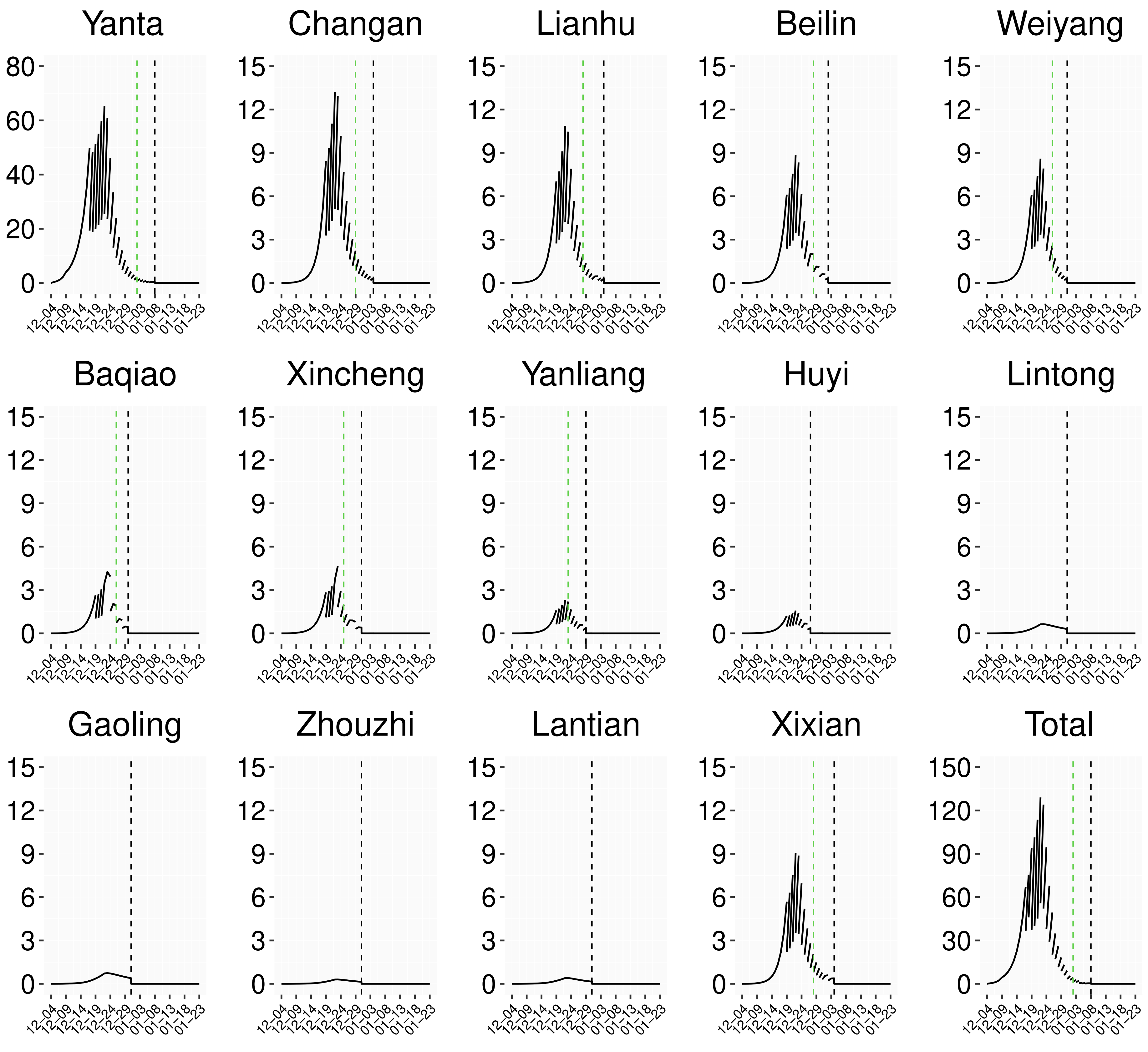
Yanta
Changan
Lianhu
Beilin
Weiyang
Baqiao
Xincheng
Yanliang
Huyi
Lintong
Gaoling
Zhouzhi
Lantian
Xixian
Total
0
20
40
60
80
3
6
9
12
15
30
60
90
120
150
12−04
12−09
12−14
12−19
12−24
12−29
01−03
01−08
01−13
01−18
01−23
