# Supplementary material for: The optimal spatially-dependent control measures to effectively and economically eliminate emerging infectious diseases
Source: PLoS Comput Biol. 2024 Oct 7;20(10):e1012498. doi: 10.1371/journal.pcbi.1012498 (PMC11486435; doi:10.1371/journal.pcbi.1012498)
Supplement: S1 Appendix — This Appendix provides details of numerical simulation algorithm of continuous time Markov chain, details of the simulated annealing algorithm that we used to solve the optimal control problem, and supplementary illustrasions and simulation to some results in the main text. (PDF) [file pcbi.1012498.s003.pdf]

S1 Appendix

Contents

1 Population mobility modelling **2**

2 The prior distribution of parameters in the epidemic model **3**

3 Estimation of the recovery rate in the epidemic model **4**

4 The control measures implemented during the outbreak in Xi'an City **5**

5 The fit of the mobility model to the data **6**

6 Effects of timely implementation of control measures on epidemic **7**

7 Determine the starting time of control measures in the simulation of the impact of transmissibility parameters on the epidemic **8**

8 Simulation algorithm of CTMC model **9**

9 Algorithm for solving the optimal control problem **10**

1. Population mobility modelling

Patch model of population mobility

We establish the differential equation system for regional population which reflects the movement of population across regions. We first adopt the patch model (1) in [1] to represent population in different regions. Since the high dimension of this model would lead to difficulties in subsequent simulations, we reduce it to Eq (10) which is used as the regional population model throughout the article.

Specifically, let v be the number of regions and $N_{ij}(t)$ be the number of residents of region i who are present in region j at time t. Let $N_i(t)$ be the population in region i at time t and N_i^r be the residents of region i, then $N_i(t) = \sum_{k=1}^{v} N_{ki}(t)$ and $N_i^r = \sum_{k=1}^{v} N_{ik}(t)$. Here we assume that the residence of any individual is fixed, so N_i^r is constant. From [1], for $i, j = 1, ..., v$ and $j \neq i$ we have

$$\begin{cases} \dfrac{dN_{ii}}{dt} = \displaystyle\sum_{k=1}^{v} r_{ik} N_{ik} - g_i N_{ii}, \\ \dfrac{dN_{ij}}{dt} = g_i m_{ij} N_{ii} - r_{ij} N_{ij}. \end{cases} \tag{1}$$

Where $g_i \geq 0$ is the rate that residents of region i leave this region, with a fraction $m_{ij} \geq 0$ going to region j. Here $\sum_{j=1}^{v} m_{ij} = 1$ and $m_{ii} = 0$. And r_{ij} is the rate that residents of region i who are in region j return home to region i (with $r_{ii} = 0$).

In the following we simplify model (1) to reduce the computational complexity of numerical simulations. From system (1) we have

$$\frac{dN_i}{dt} = -\sum_{k=1}^{v} r_{ki} N_{ki} - g_i N_{ii} + \sum_{k=1}^{v} g_k m_{ki} N_{kk} + \sum_{k=1}^{v} r_{ik} N_{ik}. \tag{2}$$

Let $\overline{N}_{ij}$ be the equilibrium of system (1) for $i, j = 1, 2, ..., v$, Eq (2) gives

$$\frac{\overline{N}_{ij}}{\overline{N}_{ii}} = \frac{g_i m_{ij}}{r_{ij}}, \tag{3}$$

Let $\varphi_{ij} = \frac{g_i m_{ij}}{r_{ij}}$ for $i \neq j$ and $\varphi_{ii} = 1$, $\varphi_i = \sum_{k=1}^{v} \varphi_{ik}$. By (3) we have

$$N_i^r = \sum_{k=1}^{v} \overline{N}_{ik} = \overline{N}_{ii} \sum_{k=1}^{v} \varphi_{ik} = \overline{N}_{ii} \varphi_i, \tag{4}$$

and the equilibrium of N_i can be obtained as

$$\overline{N}_i = \sum_{k=1}^{v} \overline{N}_{ki} = \sum_{k=1}^{v} \varphi_{ki} \overline{N}_{kk} = \sum_{k=1}^{v} \frac{\varphi_{ki}}{\varphi_k} N_k^r. \tag{5}$$

It is easy to obtain the equivalent form of Eq (2):

$$\begin{aligned} \frac{dN_i}{dt} &= -\frac{\sum_{k=1}^{v} r_{ki} N_{ki}}{N_i} N_i - \frac{g_i N_{ii}}{N_i} N_i + \sum_{k=1}^{v} \frac{g_k m_{ki} N_{kk}}{N_k} N_k + \sum_{k=1}^{v} \frac{r_{ik} N_{ik}}{N_k} N_k \\ &= -\frac{\sum_{k=1}^{v} r_{ki} N_{ki} + g_i N_{ii}}{N_i} N_i + \sum_{k=1}^{v} \frac{g_k m_{ki} N_{kk} + r_{ik} N_{ik}}{N_k} N_k. \end{aligned} \tag{6}$$

Denote the population migration rate from region i to region k at time t as

$$\tau_{ik}(t) = \frac{r_{ki} N_{ki}(t) + g_i m_{ik} N_{ii}(t)}{N_i(t)}, \tag{7}$$

then Eq (2) is written as

$$\frac{dN_i}{dt} = -\sum_{k=1}^{v} \tau_{ik}(t) N_i + \sum_{k=1}^{v} \tau_{ki}(t) N_k. \tag{8}$$

Let τ_{ik} be the limit of $\tau_{ik}(t)$ as t approaches infinity, then

$$\tau_{ik} = \frac{r_{ki}\overline{N}_{ki} + g_i m_{ik}\overline{N}_{ii}}{\overline{N}_i}. \tag{9}$$

For most realistic cases, states of system (1) rapidly converge to the equilibrium [2], thus τ_{ik} is suitable to describe the population flow across regions.

By $\tau_{ik} = \lim_{t\to\infty} \tau_{ik}(t)$ we obtain the limiting equation of system (8) as

$$\frac{dN_i}{dt} = -\sum_{k=1}^{v} \tau_{ik} N_i + \sum_{k=1}^{v} \tau_{ki} N_k, \tag{10}$$

which is the final form of the equation of regional population to model the mobility across regions.

Parameterization of mobility rate

Data on population mobility across regions is not available, so the parameter τ_{ik} can not be estimated directly. In order to apply our model to real problems, we need to estimate τ_{ik} from available data. To do this, we use the gravity model [3], and define the commuting flow w_{ij} of residents of region i to region j as

$$w_{ij} = g_i m_{ij} N_i^r. \tag{11}$$

By the gravity model of migration we can express w_{ij} as

$$w_{ij} = D\frac{G_i^a G_j^b}{f(d_{ij})}, \tag{12}$$

which is a geographical model used to predict the degree of migration interaction between two places [3]. Variable G_k represents the importance of any region k and d_{ij} is the distance between two regions i and j. Here the importance of a region can be measured in terms of population numbers, gross domestic product, or other appropriate variables.

We set G_i in formula (12) to be GDP of region i. As in [4], we take f as

$$f(d) = e^{d/82}, \tag{13}$$

and parameters $a = 0.46$, $b = 0.64$. Set $g_i m_{ij} = \frac{w_{ij}}{N_i^r}$ and $r_{ij} = 3$, then population migration rate τ_{ij} can be calculated according to formula (9) and (3)-(5), and the only unknown parameter to be estimated in the expression of τ_{ij} is D, which is estimated using the Bayesian method described in Parameters estimation section in the main text.

2. The prior distribution of parameters in the epidemic model

Let $Normal(a, b)$ be the normal distribution with mean a and standard deviation b, $Unif(a, b)$ be the uniform distribution on interval $[a, b]$. The prior distributions for the basic reproduction number $R_{0,i} := \frac{\beta_i}{\gamma}$ of region i is

$$R_{0,i} \sim Normal(2, \frac{2}{3})$$

for i = Lintong, Gaoling, Zhouzhi, Lantian and

$$R_{0,i} \sim Normal(6.5, \frac{1}{3})$$

for other regions.

The prior distributions for the false-negative rate of nucleic acid screening f is

$$f \sim Normal(0.35, 0.035) \cdot Unif(0, 1).$$

The prior distributions for reduction in contact rate after lockdown ϵ_i in region i is

$$\epsilon_i \sim Normal(0.75, 0.05) \cdot Unif(0, 1)$$

for any i.

Non-informative prior distribution is chosen for other parameters. The prior distribution $p(\boldsymbol{\theta})$ for unknown parameters $\boldsymbol{\theta}$ is the product of the prior distributions for the above parameters. In addition, we assume all parameters in the epidemic model to be nonnegative, hence $p(\boldsymbol{\theta}) = 0$ if any component of $\boldsymbol{\theta}$ is less than 0.

3. Estimation of the recovery rate in the epidemic model

From [5], the generation time (GI) of the SARS-CoV-2 Delta variant is taken to be the Gamma distribution with a mean of 4.7 days and a standard deviation of 3.3 days, and its probability density function is denoted as g_{GI}. Taking the incubation period as 4.4 days [6], and assuming that the latent period is two days shorter than the incubation period, we have $\sigma = \frac{1}{4.4-2} = 0.417$.

The intrinsic generation-interval of the epidemic model (4) in the main text is $T_{GI} = T_\sigma + T_\gamma$, where the random variables T_σ, T_γ denote the intrinsic latent period and the intrinsic infectious period of model (4), and T_σ, T_γ follow exponential distribution with mean $\frac{1}{\sigma}$, $\frac{1}{\gamma}$, respectively [7]. It is easy to obtain the probability density function of the random variable T_{GI}:

$$p_{GI}(t|\gamma) = \begin{cases} \int_0^t p_\sigma(x) p_\gamma(t-x) dx, & t \geq 0, \\ 0, & t < 0. \end{cases} \tag{14}$$

here for $\xi = \sigma, \gamma$,

$$p_\xi(x) = \begin{cases} \xi e^{-\xi x}, & x \geq 0, \\ 0, & x < 0. \end{cases} \tag{15}$$

is the probability density function of the exponential distribution with the mean of $\frac{1}{\xi}$. We fitted the model intrinsic generation time to the generation time distribution from the literature, and took the recovery rate γ as

$$\gamma = \arg\min_{z>0} \sum_{t=0}^{100} [p_{GI}(0.1t|z) - g_{GI}(0.1t)]^2. \tag{16}$$

The point estimate is $\gamma = 0.432$. In (16) the interval need to be small to ensure that the fitting is precise and we chose the interval length to be 0.1 which produced a good fitting result. The corresponding generation time distribution of model (4) and the fitting is shown in Fig 3.

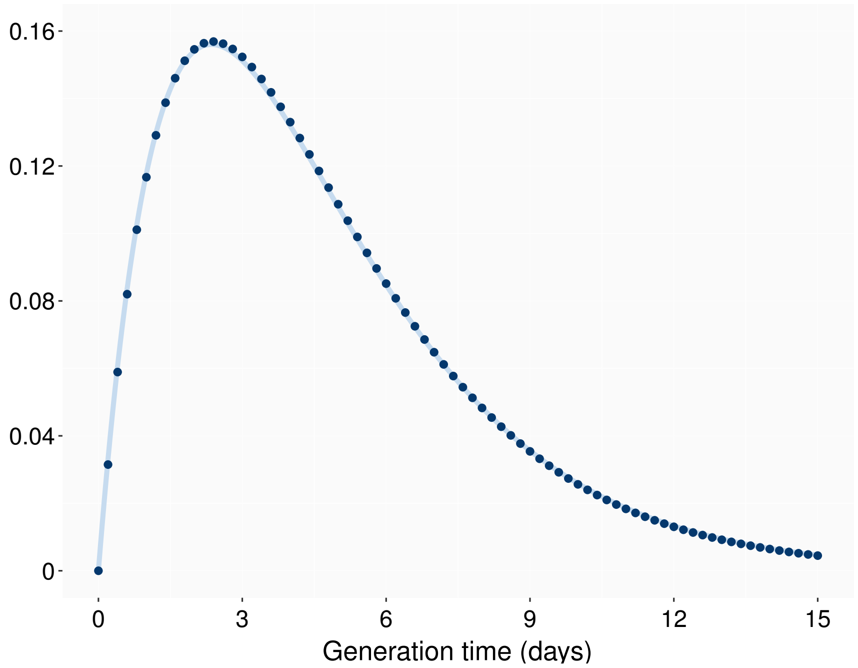

Figure 1: Probability density function of the generation interval. The line indicates the intrinsic generation interval of the epidemic model and dots indicates Gamma distribution with mean of 4.7 days and standard deviation of 3.3 days.

4. The control measures implemented during the outbreak in Xi'an City

The control measures in Xi'an during the outbreak were implemented in several stages. and use this division to determine the earliest time that each control measure can be implemented in the numerical simulation. We assume that the index case appears at the time t_0 (we choose $t_0 = 0$) in a region and the outbreak ends before the time t_f. A control measure c is implemented at t^c for $c = p, n, b, l$. Based on the actual situation, we divide the time from the importation of the index case to the end of the epidemic into the following phases:

1. Hidden transmission: The period between the initial time $t_0 = 0$ of the epidemic and the confirmation time of the index case (denoted by t^p). The outbreak grows exponentially during this period without any control measures ($u^c(t) \equiv 0$ for $c = p, n, b, l$, $t \in [t_0, t^p)$);

2. Precise control: The period between the confirmation time of the index case t^p and the starting time of large-scale control measures (namely $\min\{t^n, t^b, t^l\}$). During this period only precise control measures such as contact tracing is , but no large-scale control measures take effect ($u^c(t) \equiv 0$ for $c = n, b, l$, $u^p(t) \equiv 1$, $t \in [t^p, \min\{t^n, t^b, t^l\})$);

3. Large-scale control: The period between the start of large-scale control and the end of the epidemic ($t \in [\min\{t^n, t^b, t^l\}, t^f]$). For $c = n, b, l$ the control measure c can be implemented from t^c ($u^c(t)$ can be 0 or 1 for $c = n, b, l$ and $t \in [t^c, t_f]$, $u^p(t) \equiv 1$ for $t \in [t^p, t_f]$).

Starting from a positive initial condition, the state variables of our epidemic model (4) in the main text can only converge to zero asymptotically, rather than become zero in finite time. Therefore we propose a vector-value function G to determine whether to set infected population E, I to zero according to the state of the system. Specifically let $E_i = E_i^s + E_i^m$, $I_i = I_i^s + I_i^m$, we take

$$G(i, t, E_i(t), I_i(t)|\eta_{zero}) = \begin{cases} (0,0), & (t, E_i(t), I_i(t)) \in \Delta_i(\eta_{zero}) \\ (E_i(t), I_i(t)), & (t, E_i(t), I_i(t)) \notin \Delta_i(\eta_{zero}) \end{cases}$$

for any region i and time t, where

$$\Delta_i(\eta_{zero}) := (\Delta_{i1} \cap \Delta_{i2} \cap \Delta_{i3})(\eta_{zero})$$

and

$$\begin{aligned} \Delta_{i1}(\eta_{zero}) :=& \{(t, E, I) : \ \text{Pois}(0, E + I) > \eta_{zero}\}, \\ \Delta_{i2}(\eta_{zero}) :=& \{(t, E, I) : \ t > t_i^p\}, \\ \Delta_{i3}(\eta_{zero}) :=& \{(t, E, I) : (1 - u_i^b(t)) \sum_{j \neq i} (1 - u_j^b(t)) \chi_{\{\text{Pois}(0, (E_j + I_j)(t)) < \eta_{zero}\}} = 0\}. \end{aligned}$$

Where $\text{Pois}(0, x)$ is the probability that the Poisson-distributed random variable with mean x taking the value 0, and χ_A is the index function of set A which takes 1 when A holds and 0 otherwise.

In the construction of function G, the set $\Delta_{i1}(\eta_{zero})$ indicates that in region i the number of cases who haven't been isolated is less than a 'zero threshold' which is determined by the parameter η_{zero}. The set $\Delta_{i2}(\eta_{zero})$ indicates that region i is out of the stage of hidden transmission at time t. The set $\Delta_{i3}(\eta_{zero})$ indicates there is no region that has population mobility with region i and in which the number of cases who haven't been isolated is greater than 0. We set $(E_i(t), I_i(t)) = G(i, t, E_i(t), I_i(t)|\eta_{zero})$ at each integer time t, so that if the system states satisfies conditions $\Delta_{i1}(\eta_{zero})$, $\Delta_{i2}(\eta_{zero})$ and $\Delta_{i3}(\eta_{zero})$ then number of cases out of isolation area in region i will get zero at time t.

To determine the value of η_{zero} in function G, we denote the latest isolation time of cases of region i in observed data as $t_{i,d}^{zero} = \max\{t : x_i(t) > 0\}$ (shown in the figure of daily new isolated cases in the main text). We solve model (4) under parameters $\boldsymbol{\theta}$ and control variables $\boldsymbol{U}_0$, with mapping $(E_i(t), I_i(t)) \longrightarrow G(i, t, E_i(t), I_i(t)|\eta)$ at each intger time t, to obtain a model fit value $\mu_i(t; \boldsymbol{\theta}, \boldsymbol{U}_0)|\eta$ for observation data of new cases $x_i(t)$. Let $t_i^{zero}(\eta) := \max\{t : \mu_i(t; \boldsymbol{\theta}, \boldsymbol{U}_0|\eta) > 0\}$, then η_{zero} is taken as

$$\eta_{zero} = \underset{\eta \in (0,1)}{\arg\min} \sum_{i=1}^{v} (t_{i,d}^{zero} - t_i^{zero}(\eta))^2. \tag{17}$$

5. The fit of the mobility model to the data

We use

$$\frac{dN_i}{dt} = -\sum_{k=1}^{v} \tau_{ik} N_i + \sum_{k=1}^{v} \tau_{ki} N_k. \tag{18}$$

to model the population mobility across regions and estimated parameters τ_{ik} by using the gravity model. In order to verify the rationality of the estimated value of population mobility matrix $(\tau_{ik})_{n\times n}$, We obtain the equilibrium $\overline{N}_i$ of system (18) and compare it with the number of residents N_i^r. For most practical cases, $\overline{N}_i$ should be close to N_i^r.

Let the right side of system (18) to be 0, we have the equations of equilibriums

$$A\vec{x} = \vec{b}, \tag{19}$$

where the entry in the i-th row and j-th column of coefficient matrix $A = (a_{ij})_{v\times v}$ is

$$a_{ij} = \begin{cases} 1, & i = 1,\ 1 \le j \le v \\ -\tau_{ij}, & 1 \le i, j \le v,\ i \ne j \ and \ i \ne 1 \\ \sum_{k=1}^{v} \tau_{ki}, & 1 \le i, j \le v,\ and \ i = j \end{cases} \tag{20}$$

vector $\vec{b} = (b_1, ..., b_v)$ satisfies $b_1 = \sum_{i=1}^{v} N_i^r$and $b_2 = b_3 = ... = b_v = 0$. $\overline{N}_i$ is derived by solving system 19 for $\vec{x}$ and is shown in Fig 2. And max $|\overline{N}_i - N_i^r| = 1645$, which is negligible compared to the magnitude of the population of districts and counties.

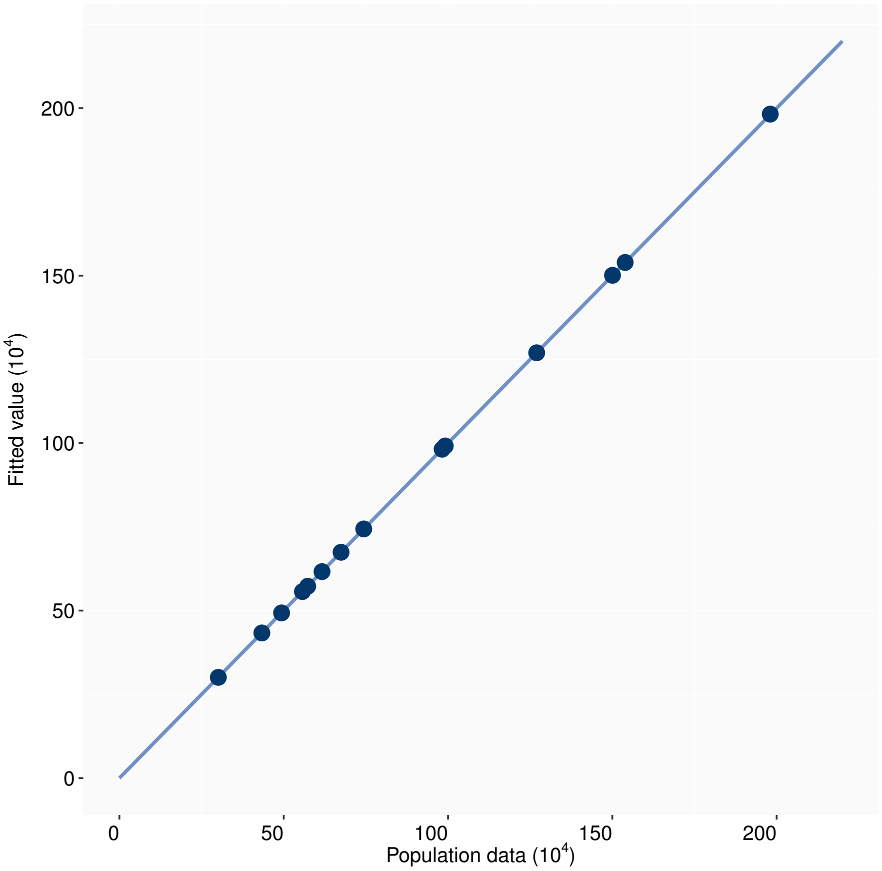

Figure 2: The number of residents (dots) and the equilibrium of the population (broken lines) in each district and county of Xi'an City.

6. Effects of timely implementation of control measures on epidemic

In the discussion of the effect of control measures' starting time on the epidemic, we find that a few days in advance of implementing regional border closure does not impact the epidemic significantly, but a large enough number of days in advance of regional border closure causes a significant decrease in the number of infections. We show the graphic of $I_i(t) := \sum_{\omega=m,s} I_i^m + I_i^s$ for regions $i = 1, 2, ..., v$ and $d^b = 0, -5, -12$ in Fig 3. Here d^b is the days of change in the starting time of border closure and negative values represent advancement. From Fig 3 we can see that the phenomenon is due to that early enough implementation of regional border closure prevents the importation of cases from Yanta District to districts in which local transmission has occurred under the baseline control scheme.

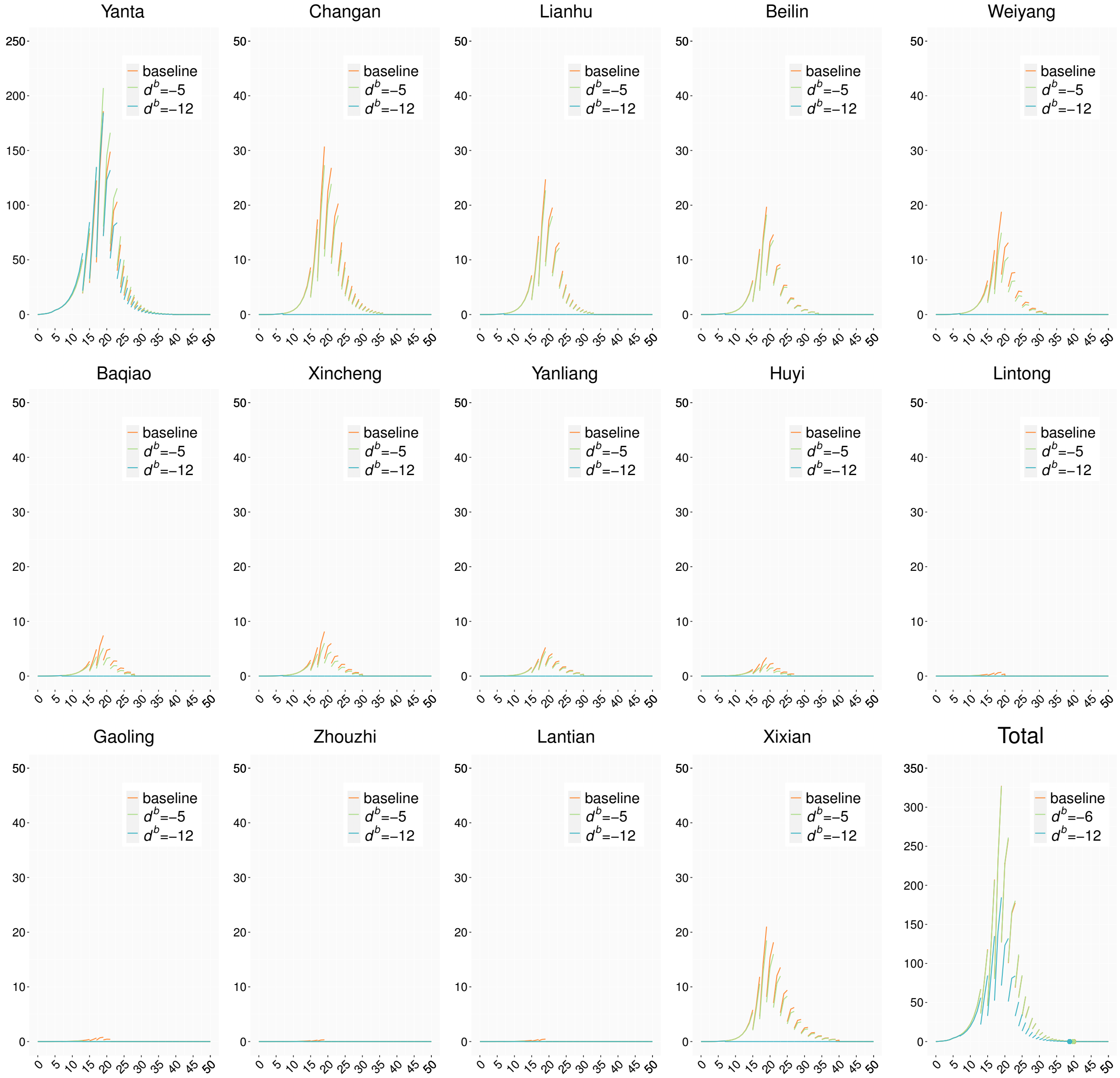

Figure 3: Number of infected individuals $I_i(t)$ in the infectious period in region i under different control schemes. 'baseline' represents baseline control scheme, d^b is the change in the starting time of border closure relative to baseline, with negative values representing advancement.

7. Determine the starting time of control measures in the simulation of the impact of transmissibility parameters on the epidemic

In practice, changes in transmissibility parameters can lead to changes in the rate of epidemic size growth, diseases that transmit faster produce more infections in shorter time, hence the outbreak is detected earlier by local governments. Therefore, we determine the earliest time when control measures can be implemented under each set of parameters based on the number of infections or confirmed cases in the discussion of the impact of transmissibility parameters on the outbreak.

Remember that the baseline control scheme

$$\boldsymbol{U}_0 := \{u^c_{i,0}(t) : i = 1, ..., v;\ c = p, n, b, l;\ t \geq 0\}, \tag{21}$$

is taken as control measures implemented in Xi'an City from December 2021 to January 2022. Then the earliest time that control measure c under the baseline control scheme implemented in Xi'an City is

$$t^c := \min\{t :\ u^c_{i,0}(t) = 1,\ i = 1, 2, ..., v.\},\ c = p, l, b, n. \tag{22}$$

We denote the cumulative number of infections and confirmed cases at time t under the parameter set par as $C^1_{par}(t)$, $C^2_{par}(t)$, respectively. The end time of convert transmission stage under parameter set par is taken to be

$$t^p_{par} = \min\{t \geq 0 :\ C^1_{par}(t) \geq C^1_{base}(t^p)\}, \tag{23}$$

here the subscript $base$ indicates baseline parameters.

The earliest time when nucleic acid screening can be implemented is taken as

$$t^n_{par} = \min\{t \geq 0 :\ C^2_{par}(t) \geq C^2_{base}(t^n)\}, \tag{24}$$

and the earliest time when lockdown and regional border closure can be implemented is taken as

$$t^b_{par} = t^l_{par} = \min\{t \geq 0 :\ C^2_{par}(t) \geq C^2_{base}(t^l)\}. \tag{25}$$

Denote the control scheme under parameter set par as

$$\boldsymbol{U}_{par} = \{u^c_{i,par}(t) : i = 1, ..., v;\ c = p, n, b, l;\ t \geq 0\}, \tag{26}$$

then we take

$$u^c_{i,par}(t) = u^c_{i,0}(t + (t^c - t^c_{par})) \tag{27}$$

for $i = 1, 2, ..., v.$ and $c = p, l, b, n$.

8. Simulation algorithm of CTMC model

In this paper, we mainly use ODE system (4) defined in the main text to simulate disease transmission. In addition, the continuous time Markov chain (CTMC) model corresponding to model (4) can more realistically simulate the development of the epidemic when the number of cases is small [8]. Assuming that the index case appears at time $t = 0$, we define the Markov chain

$$\boldsymbol{X}(t) = (X_1^S, X_1^{E^m}, X_1^{E^s}, X_1^{I^m}, ..., X_1^R, X_2^S, ..., X_2^R, ..., X_n^S, ..., X_n^R)(t), \quad t \geq 0, \tag{28}$$

here any element X_i^K takes a non-negative integer value and represents the random variable for system variable K_i in the epidemic model. The transition rates of the CTMC epidemic model (28) is shown in Table 8.

Table 1: State transitions and rates

Event	Transition	Transition rate
Move of K_i to K_j for $K = S, E, I, R$.	$X_i^K \to X_i^K - 1, \quad X_j^K \to X_j^K + 1$	$m_{ij}(\boldsymbol{u})X_i^K$
Infection of S_i.	$X_i^S \to X_i^S - 1, \quad X_i^{E^\omega} \to X_i^{E^\omega} + 1$	$q^\omega(1 - \varepsilon_i u_i^l)\beta_i \frac{X_i^S X_i^I}{X_i^{Nf}}$
Progression of E_i^ω to I_i^ω.	$X_i^{E^\omega} \to X_i^{E^\omega} - 1, \quad X_i^{I^\omega} \to X_i^{I^\omega} + 1$	$\sigma X_i^{E^\omega}$
Progression of $C_i^{E,\omega}$ to $C_i^{I,\omega}$.	$X_i^{C^{E,\omega}} \to X_i^{C^{E,\omega}} - 1, \quad X_i^{C^{I,\omega}} \to X_i^{C^{I,\omega}} + 1$	$\sigma X_i^{C^{E,\omega}}$
Quarantine of K_i for $K = E^\omega, I^\omega$.	$X_i^K \to X_i^K - 1, \quad X_i^{C^K} \to X_i^{C^K} + 1$	$u_i^p L_i^K X_i^K$
Quarantine of I^ω.	$X_i^{I^\omega} \to X_i^{I^\omega} - 1, \quad X_i^{C^{I,\omega}} \to X_i^{C^{I,\omega}} + 1$	$\delta_i X_i^{I^\omega}$
Recovery of K_i for $K = I^m, C^{I,m}$.	$X_i^K \to X_i^K - 1, \quad X_i^R \to X_i^R + 1$	$\gamma^m X_i^K$
Hospital admission of K_i for $K = I^s, C^{I,s}$.	$X_i^K \to X_i^K - 1, \quad X_i^H \to X_i^H + 1$	$\gamma^s X_i^K$
Discharge of H_i.	$X_i^H \to X_i^H - 1, \quad X_i^R \to X_i^R + 1$	$\gamma^H X_i^H$

We use the Gillespie algorithm to generate the sample path of the CTMC model (28) between two adjacent impulsive moments:

Step 0. Initialize time $t = T_0$ and system state $\boldsymbol{x}_0 = \boldsymbol{x}(T_0)$. We denote the time of the $l-$th event occurring after the time $t = T_0$ as $T_l, l = 1, 2, ...$

Step 1. For system state $\boldsymbol{x}(t)$ at time $t = T_l$ $(l = 0, 1, 2, ...)$, calculate transition rate $a_j(\boldsymbol{x}(t))$ of event j and the sum of transition rates of all events $a(\boldsymbol{x}(t)) = \sum_j a_j(\boldsymbol{x}(t))$ according to Table 8.

Step 2. Sample a random number ς from the exponential distribution $Exp(a(\boldsymbol{x}(t)))$ and a random number r from the uniform distribution $Unif(0, 1)$, and take

$$\vartheta = \min\{j : \quad (\sum_{j'=1}^{j})/a(\boldsymbol{x}(t)) \geq r\}, \tag{29}$$

Let the occurring time of the next event be $t = T_{l+1} = T_l + \varsigma$ and the next event to occur be ϑ. Then update the system state $\boldsymbol{x}(t)$.

Step 3. Return to Step 1 until $T_L \geq t_T := \min\{T : T > T_0 \ \ and \ \ T \in \{t_s\}_{s=1,2,...}\}$ holds.

For any impulsive time t_s, we make the state of the system (28) change in the following way:

$$\begin{cases} X_i^{I^\omega}(t_s) &= X_i^{I^\omega}(t_s^-) - Y_i(t_s), \\ X_i^{C^{I,\omega}}(t_s) &= X_i^{C^{I,\omega}}(t_s^-) + Y_i(t_s), \\ X_i^K(t_s) &= X_i^K(t_s^-), \quad K \neq I^\omega, C^{I,\omega}. \end{cases} \tag{30}$$

Where the random variable $Y_i(t_s)$ follows the binomial distribution $B(X_i^{I^\omega}(t_s^-), u_i^n(t_s)(1-f))$, and $\{Y_i(t_s)\}_{i,s\geq 1}$ are independent of each other.

To obtain the results in the main text Fig 4, we repeated the simulation of CTMC 500 times for each scenario, in each simulation we recorded the time of the first appearance of infected individuals and the number of imported cases in each region. A simulation was terminated when the number of infected individuals in all regions had reached zero or when border closure had been implemented in all regions.

9. Algorithm for solving the optimal control problem

We abbreviate the disease transmission model used in the optimal control problem in the main text as

$$\begin{cases} \boldsymbol{y}'(t) = \boldsymbol{f}(\boldsymbol{y}(t), \boldsymbol{U}(t)), & t \notin \{t_s\}_{s=1,2,...} \\ \boldsymbol{y}(t_s) = \boldsymbol{g}(\boldsymbol{y}(t_s^-), \boldsymbol{U}(t_s)), & s = 1, 2, ... \end{cases} \tag{31}$$

where

$$\boldsymbol{U}(t) = \{u_i^c(t), u_i^b(t), u_i^n(t)\}_{i=1,...,v}, \quad t \in [0, t_f], \tag{32}$$

is the set of control variables for all regions, v is the number of regions, and the final time t_f is an integer. By our assumptions on the form of control variables, for any $l = c, b, n$ and $i = 1, 2, ..., v$, the function u_i^l is a step function on $[0, t_f]$ with integers as discontinuities, and u_i^l takes the value 0 or 1. Thus the control function $\boldsymbol{U}$ on $t \in [0, t_f]$ can be represented by a matrix of dimension $t_f \times 3v$. For $j = 1, ..., t_f$, $i = 1, ..., v$, the entries in the j-th row and $(3i-2)$-th to $3i$-th column of the matrix $\boldsymbol{U}$ are $u_i^c(j-1), u_i^b(j-1), u_i^n(j-1) \in \{0, 1\}$.

In addition to the assumptions on the form of the control variables, we also require that control measures $\boldsymbol{U}$ should make the solution $\boldsymbol{y}$ of the disease transmission model (31) satisfies constraints we set. We denote these constraints as $\boldsymbol{h}(\boldsymbol{y}(t)) \le \boldsymbol{0}$. Then the optimal control problem in the main text can be abbreviated as

$$\begin{aligned} \min_{\boldsymbol{u^c}, \boldsymbol{u^b}, \boldsymbol{u^n}} & \left(J(\boldsymbol{U}) = \sum_{i=1}^{v} \sum_{l=c,b,n} cost_i^l \right), \\ \text{s.t.} \quad & system \ (31), \\ & \boldsymbol{h}(\boldsymbol{y(t)}) \le \boldsymbol{0}, \quad t \in [0, t_f]. \end{aligned} \tag{33}$$

We solve the above optimal control problem using the simulated annealing algorithm [9]. First, according to the assumption, for any $i = 1, ..., v$, we have $u_i^n(t) \equiv 0$ when $t < t_i^n$, and $u_i^c(t), u_i^b(t) \equiv 0$ when $t < t_i^c$, where t_i^n, t_i^c are the starting times of the stage of universal nucleic acid screening and the Stage of lockdown, respectively. Except these elements which are assumed to be zero, for any entry $u_i^l(j-1)$ of the matrix $\boldsymbol{U}$, we sample a random number from the Bernoulli distribution

$$f(k; p) = \begin{cases} p, & k = 1, \\ 1 - p, & k = 0. \end{cases} \tag{34}$$

as the initial value of $u_i^l(j-1)$, which gives the initial value $\boldsymbol{U}_0$ of the control matrix $\boldsymbol{U}$. Where p is sufficiently large to make the constraint $\boldsymbol{g}(\boldsymbol{U}) \le \boldsymbol{0}$ holds, and in this paper we take $p = 0.9$. The algorithm for solving (33) is as follows:

Step 0. Initialize the temperature as $c = c_0$ and the control variable as $\boldsymbol{U} = \boldsymbol{U}_0$.

Step 1. For the temperature $c = c_k$, repeat steps 1.1-1.2 for L_k times:

1.1. Randomly select an entry $u_i^l(j-1)$ of the control matrix $\boldsymbol{U}$ that can take the value of 1 and change its value to $1 - u_i^l(j-1)$ to get a new control matrix $\boldsymbol{V}$. Then plug $\boldsymbol{V}$ into system (31) to solve for the solution $\boldsymbol{y}$. If the constraint $\boldsymbol{h}(\boldsymbol{y(t)}) \le \boldsymbol{0}$ holds, then proceed to step 1.2, otherwise obtain the matrix $\boldsymbol{V}$ again in the same way.

1.2. If $J(\boldsymbol{V}) \le J(\boldsymbol{U})$ holds, then let $\boldsymbol{U} = \boldsymbol{V}$. Otherwise, sample a random number $rand$ from the uniform distribution $U(0, 1)$. When

$$exp\left(\frac{J(\boldsymbol{U}) - J(\boldsymbol{U})}{\lambda \cdot c_k}\right) > rand \tag{35}$$

holds, set $\boldsymbol{U} = \boldsymbol{V}$, otherwise set $\boldsymbol{U} = \boldsymbol{U}$.

Step 2. Set $c_k = \omega \cdot c_k$ and $k = k + 1$. If $c_k \ge c_{end}$, return to Step 1, if $c_k < c_{end}$ then stop.

In the above steps we take the initial temperature $c_0 = 1 \times 10^4$, the minimum temperature $c_{end} = 0.1$, the number of searches at each temperature $L_k = 500$, the coefficient $\lambda = 3.5 \times 10^5$ in (35), and the temperature drop coefficient $\omega = 0.9$.

The convergence of the algorithm is shown in Fig 4, The horizontal axis represents the number of iterations of the algorithm, the vertical axis of the left figure represents the difference between the objective function of the current iteration and the last iteration, and the vertical axis of the right figure represents the objective function value.

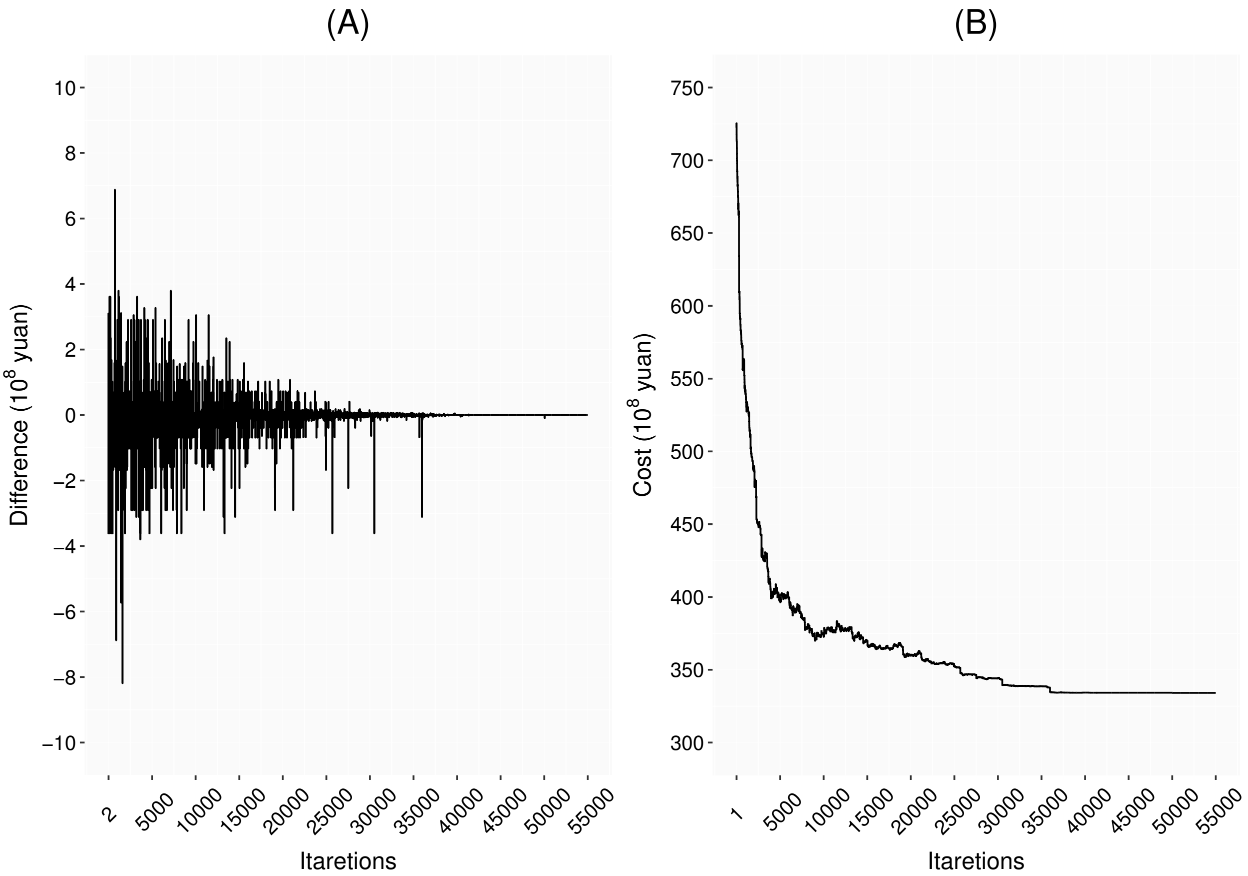

Figure 4: The convergence of the simulated annealing algorithm.

References

[1] van den Driessche P. Spatial Structure: Patch Models. In: Brauer F, van den Driessche P, Wu J, editors. Mathematical Epidemiology. Berlin: Springer; 2008. pp. 179–189.

[2] Keeling MJ, Rohani P. Estimating spatial coupling in epidemiological systems: a mechanistic approach. Ecology Letters. 2002;5(1):20–29.

[3] Rodrigue JP. The geography of transport systems. 3rd ed. Routledge; 2013.

[4] Balcan D, Colizza V, Gonçalves B, Hu H, Ramasco JJ, Vespignani A. Multiscale mobility networks and the spatial spreading of infectious diseases. Proceedings of the National Academy of Sciences. 2009;106(51):21484–21489.

[5] Hart WS, Miller E, Andrews NJ, Waight P, Maini PK, Funk S, et al. Generation time of the alpha and delta SARS-CoV-2 variants: an epidemiological analysis. The Lancet Infectious Diseases. 2022;22(5):603–610.

[6] Zhang M, Xiao J, Deng A, Zhang Y, Zhuang Y, Hu T, et al. Transmission dynamics of an outbreak of the COVID-19 Delta variant B. 1.617. 2—Guangdong Province, China, May–June 2021. China CDC Weekly. 2021;3(27):584.

[7] Champredon D, Dushoff J, Earn DJD. Equivalence of the Erlang-distributed SEIR epidemic model and the renewal equation. SIAM Journal on Applied Mathematics. 2018;78(6):3258–3278.

[8] Allen LJS. Stochastic population and epidemic models. Berlin: Springer; 2015.

[9] Burke EK, Kendall G. Search methodologies: introductory tutorials in optimization and decision support techniques. 2nd ed. Berlin: Springer; 2014.
