# Supplementary material for: The optimal spatially-dependent control measures to effectively and economically eliminate emerging infectious diseases
Source: PLoS Comput Biol. 2024 Oct 7;20(10):e1012498. doi: 10.1371/journal.pcbi.1012498 (PMC11486435; doi:10.1371/journal.pcbi.1012498)
Supplement: Attachment — Submitted filename: response ro reviewers.docx [file pcbi.1012498.s004.docx]

Dear Editor,

We are very grateful to the referees for their thorough review and constructive comments. We have revised the manuscript accordingly. Please see the point-by-point response letter below. The entire article has been polished and changes made to the content in the manuscript are marked in blue. The readability of our manuscript, especially to a broader audience, has been greatly improved by these revisions.

Sincerely,

Junling Ma

On behalf of the authors

**Response to reviewers 1' comments**:

Comment (1): The Introduction and Literature Review sections require a more detailed rewrite. The introduction should furnish a comprehensive summary of relevant prior works, with explicit references pinpointing the gaps in the existing literature.

Response: Thank you for your constructive comments and suggestions. We have added contents in the introduction to summarize the relevant literature. Please see lines 23—49.

Comment (2): The authors are advised to reconsider or redesign Figures 2 to 6. The red background in each figure appears aesthetically unpleasing and may hinder comprehension. A modification or redraw addressing the visual aspect would enhance the overall clarity of the figures.

Response: We have redesigned all figures.

**Response to reviewers 2' comments**:

Comment (1): The Bayesian parameter estimation model entails the fitting of numerous parameters. A more extensive discussion regarding this process, along with an assessment of the outcomes in relation to existing models or literature, would enhance the comprehensiveness of the paper. While some parameters may not directly correlate with the specific context, it is still worthwhile to explore any relevant comparisons.

Response: Thank you for your constructive comments and suggestions. We have clarified parameter estimation. Please see lines 146—175. We also compared our results with the estimated values in the literature, and showed their agreements in lines 427—439.

Comment (2): The authors acknowledge that the economic costs portrayed in the model are somewhat high-level. Given that this aspect constitutes a significant component of the paper's objectives, providing additional details on the sources of these costs, their units, and any adjustments made would be beneficial. What would be the implications of accounting for costs associated with hospitalisation and deaths?

Response: We provided a more detailed description of the construction of epidemic control cost in lines 190—208, and a discussion of the limitations and extensions of our cost function to include other costs such as deaths in lines 481—489.

Comment (3): Several figures could benefit from refinement to enhance clarity and ease of interpretation. For example:

• While most journal readers will likely be able to interpret chunks of the Figure 1 diagram, some may be difficult to interpret without more information, especially as the model is only described later. For example, a few of the states are not easy to interpret without finding where the main text refers to them. I suggest adding sufficient information to understand the diagram. Perhaps moving the diagram down to the model section also helps.

Response: We have revised Figure 1 and to only lead the map of Xi’an showing the districts. The flowchart has been moved to the Methods section as a new Figure 5,

• Figure 5 C3 is difficult to interpret in places. Moving the legends, perhaps to the side or bottom, or adding a white background to the legend could be helpful.

Response: We have moved the panel Figure 5C out of the figure into the new Figure 7. The figure is redrawn to avoid the overlap of legend and content.

• Fig 6 has a lot going on. Perhaps the figure can be simplified for quicker interpretation. For example, 6A could be separated into multiple pots. Perhaps there’s a clearer way to convey the message about R0, GDP and optimal control cost moving together.

Response: There was indeed some redundant information in this figure. We have redesigned it in the new Fig 8, to make it easier to follow. We have also made some improvements to other figures.

**Response to reviewers 3' comments**:

Comment (1): This manuscript is about optimal and timely spatial deployment of non-pharmaceutical interventions (NPIs) at the early stages of an outbreak of an infectious disease to prevent it from establishing. The paper would be of great relevance four years ago, but the novelty at this point in time is questionable.

Response: Thank you for your constructive comments and suggestions. Our study is retrospective. Instead of providing the optimal strategy to control the COVID-19 outbreak in Xi’an, we aim to use this outbreak as an example to develop a comprehensive modeling framework to address future emerging and re-emerging infectious disease outbreaks. The highlights of our modeling framework include a) a realistic population movement model to address population heterogeneity and region-specific control strategies, b) a reliable parameter estimation method to address population heterogeneity, and c) a flexible cost function to incorporate control measures that have different scales of socio-economic costs. We have added a discussion of these points in 412-417 for clarification.

Comment (2): The paper is solid and, as far I as I can tell without access to the code and data, is technically sound.

Response: We have made the code and data publicly available on github (<https://github.com/nidefanwang/optimal_spatially_dependent_control_measures_for_emerging_infectious_diseases.git>).

However, I do not think this paper is suitable for PLOS Comp Bio for several reasons: 1) it is written for a technical audience and not put in a larger applicable context,

Response: Indeed, the original version was very mathematical, and was not put in a larger context. We have rewritten the majority of the Introduction to address the general audience and put this work in the literature in a big picture. We have also extensively revised the Methods section to explain the concepts in detail for a general audience, and reduced the technicality to a minimum in the Methods section while retaining rigorousness and reproducibility. Much of the technical details such as the population movement model and mathematical details of incorporating control measures have been moved to the supplementary materials.

2) it covers a very narrow literature, ignoring vast areas of applications in epidemic modelling

Response: We have rewritten the literature review to include non-modeling methods such as case studies, mathematical modeling approaches to assess the effectiveness of control measures, previous work on optimal disease control measures. Please see lines 23—49.

and 3) therefore missing the novelty and the relevance. What makes a disease easily controllable (Fraser et al Science), relevance of commuters for spreading influenza in the US (Viboud et al, Science), reducing contact rates as an intervention (papers like Prem et al that do that by using contact data) are all seminal papers in the field that would provide relevant context for this work, relate it to a broader set of data and applications, and clarify what exactly this paper adds in terms of novelty.

Response: Our aim is to provide a computational tool that can be applied to the control of future emerging disease outbreaks. The reviewer provided references to modeling approaches to specific interventions, which we have cited in the Introduction, as these are important components of our modeling framework. In fact, we comprehensively integrate these methods to provide a novel framework for optimal control strategies using a retrospective study approach. This is explained in the Introduction in lines 50—62. In addition, we added a discussion of our contribution in lines 494—505.

Comment (3): Authors make a lot of assumptions on flows between patches which are scaled by GDP and distance. The following sentence has a reference to Balcan et al 2009, but these authors use mobility data to capture the flows in their multiscale mobility networks, rather than distance and GDP. There is a vast amount of literature on flows between patches (from mobility data (e.g. cellphone data, and airline traffic data, google mobility), census data (e.g. using postcodes of home and workplaces to infer flows), traffic, work place data) so it is surprising not to see any reflection on that literature in the manuscript and link it with the assumptions about the flows here. Referring to a study that analyses suitability of using GDP in scaling of gravity models would be one way to clarify this.

Response: We have moved the estimation of the migration rates into the supplementary materials. We have cited Rodrigue 2013 for gravity model. We also cited Balcan et al. 2009 to use some of their estimated parameter values of the gravity model. In the discussion of the main text, we cited Viboud et al. 2006 [21] for a gravity model using the population size, and Merler et al. 2010 [50] for a gravity model using GDP. We also discussed the other methods such as traffic data, workflow data and mobile phone data, and the reason for choosing gravity model in this framework because these data are not readily available to researchers. Please see lines 473—480. More mathematical models involving population movement has been references in line 34.

Comment (4): There are several instances of inappropriate referencing of claims in the paper, for example, citing newspaper article (ref [21]) to support effectiveness of a strategy rather than peer-reviewed analysis of effectiveness of a strategy). “Since contact tracing is only effective if the outbreak is detected early before widespread community transmission [21], ([21] People’s Daily Online. Interpretation of the new policy of epidemic prevention in Shanghai; 2022. Available from: http://health.people.com.cn/n1/2022/0319/c14739-32378995.html.) Instead, the validity of this statement should be supported by a published peer-reviewed paper or shown with own work presented in this manuscript.

Response: We agree here. Unfortunately, COVID-19 data in China are generally released by provincial and municipal in the form of press conferences, announcements, notices, and documents, and many of the data used in this paper were collected from these sources. Except for these references, we have removed unnecessary citations to non-journal articles as possible and replaced them with appropriate papers.

Comment (5): There are several technical issues that need clarifying/justifying, for example:

a) lockdown is assumed to have different impact in different locations – is this modelling adherence/ compliance/ or that different locations have different proportions of e.g. keyworkers whose work and commute patterns will be different form non-key workforce? Please elaborate this.

Response: We have added a discussion on lines 435—439 to state that the different demographic and economic conditions of regions may lead to difference in extent of implementing control measures and compliance of residents. Specifically, we made such parameters region-dependent, and estimated the impact of lockdown (parameter epsilon_i in the manuscript) by fitting the epidemic data. According to the results of parameter estimation (Table 1), although there are differences in the percentage of contact rate reduction in different regions, they are all around 70%, which is not a particularly large difference.

b) Contact trancing is inherently an individual-based process and not straightforward to capture in population-level, compartmental models. Why is a logistic decay function a suitable one for modelling rates of isolation of exposed and infectious individuals? The only reference in this paragraph (lines 147-153) is a newspaper article, which I doubt will inspire readers of Plos Comp Bio with confidence.

Response: We have clarified on lines 127—130 that the effectiveness of contact tracing is affected by the outbreak size and decreases when the number of cases exceeds the test-trace-isolate capacity of the local health authority. Hence we assume that the contact tracing rate is approximately constant within the capacity of contact tracing, starts to decrease beyond capacity, and approaches zero when the outbreak size is very large. The logistic decay function is a decreasing function with this property and is chosen to model the isolation following contact tracing.

c) Whole sections of paper (e.g. Estimation section, Optimal control section) have no references to other literature (e.g. MCMCs, Bayesian inference, modelling over-dispersion, defining a cost function and specifying an optimal control problem, using methods for minimising the cost or utility function should all refer to the relevant literature).

Response: We have explained our Bayesian formulation, the incorporation of over-dispersion on lines 161—170, the implementation details of MCMC on lines 171—175, and numerical methods to solve the optimal control problem on lines 220—222. While some parts are mainly authors’ derivation and construction, without reference to the conclusions and methods of other studies, such as the definition of the cost function and the establishment of the optimal control problem.
